# SMYD2 Inhibition Downregulates TMPRSS2 and Decreases SARS-CoV-2 Infection in Human Intestinal and Airway Epithelial Cells

**DOI:** 10.3390/cells11081262

**Published:** 2022-04-08

**Authors:** Yu-Qiang Yu, Alexandra Herrmann, Veronika Thonn, Arne Cordsmeier, Markus F. Neurath, Armin Ensser, Christoph Becker

**Affiliations:** 1 Department of Medicine 1, Friedrich-Alexander-Universität Erlangen-Nürnberg (FAU), 91052 Erlangen, Germany; yuqiang.yu@duke.edu (Y.-Q.Y.); veronika.thonn@uk-erlangen.de (V.T.); markus.neurath@uk-erlangen.de (M.F.N.); 2 Deutsches Zentrum Immuntherapie (DZI), 91052 Erlangen, Germany; 3 Department of Immunology, Duke University School of Medicine, Durham, NC 27710, USA; 4 Institute of Clinical and Molecular Virology, Friedrich-Alexander-Universität Erlangen-Nürnberg (FAU), 91052 Erlangen, Germany; alexandra.herrmann@uk-erlangen.de (A.H.); arne.cordsmeier@uk-erlangen.de (A.C.)

**Keywords:** SMYD2, TMPRSS2, SARS-CoV-2, COVID-19, BAY598, AZ505, antiviral treatment

## Abstract

The COVID-19 pandemic caused by SARS-CoV-2 has lasted for more than two years. Despite the presence of very effective vaccines, the number of virus variants that escape neutralizing antibodies is growing. Thus, there is still a need for effective antiviral treatments that target virus replication independently of the circulating variant. Here, we show for the first time that deficiency or pharmacological inhibition of the cellular lysine-methyltransferase SMYD2 decreases TMPRSS2 expression on both mRNA and protein levels. SARS-CoV-2 uses TMPRSS2 for priming its spike protein to infect target cells. Treatment of cultured cells with the SMYD2 inhibitors AZ505 or BAY598 significantly inhibited viral replication. In contrast, treatment of Vero E6 cells, which do not express detectable amounts of TMPRSS2, had no effect on SARS-CoV-2 infection. Moreover, by generating a recombinant reporter virus that expresses the spike protein of the Delta variant of SARS-CoV-2, we demonstrate that BAY598 exhibits similar antiviral activity against this variant of concern. In summary, SMYD2 inhibition downregulates TMPRSS2 and blocks viral replication. Targeting cellular SMYD2 represents a promising tool to curtail SARS-CoV-2 infection.

## 1. Introduction

The severe acute respiratory syndrome (SARS) coronavirus (CoV)-2, the causative agent of the CoV disease 19 (COVID-19), was first described in Wuhan, China, at the end of 2019 [1]. Globally, the pandemic SARS-CoV-2 outbreak has caused more than 6,000,000 deaths with over 450,000,000 confirmed cases until March 2022 (https://covid19.who.int/, accessed on 1 March 2022). SARS-CoV-2 is an enveloped, positive-sense single-stranded RNA virus of the genus Betacoronavirus. The virus shares many similarities with other highly pathogenic coronaviruses, such as SARS-CoV and MERS. The viral spike (S) glycoprotein of both SARS-CoV and SARS-CoV-2 uses angiotensin-converting enzyme 2 (ACE2) as a receptor to mediate cell entry [2,3], while MERS mediates infection by binding to the dipeptidyl peptidase 4 (DPP4) [4]. The S protein of mature SARS-CoV-2 virions consists of two non-covalently linked subunits, S1 and S2. The S1 subunit binds ACE2, whereas the S2 subunit anchors the S protein to the host cell membrane and mediates membrane fusion upon infection of the target cells (reviewed in [5]). In a priming step, the S2 protein is cleaved by host proteases at the S2’ cleavage site. Two different classes of proteases were shown to mediate S protein priming, namely the endolysosomal proteases of the cathepsin family and the plasma membrane-associated transmembrane protease serine type 2 (TMPRSS2) [6,7,8]. Additionally, the SARS-CoV-2 S protein possesses a unique furin-like cleavage site and thus is intracellularly processed by furin [8,9,10].

Although highly effective vaccines are now available, a broad repertoire of specific antiviral drugs and therapeutics for the prevention and treatment of COVID-19 are still urgently needed. To date, the only Food and Drug Administration (FDA)- and European Medicines Agency (EMA)-approved antiviral drugs are remdesivir (GS-5734, veklury) and paxlovid (PF-073321332 and ritonavir). Remdesivir must be administered intravenously, and its early administration has been shown to prevent progression to severe COVID-19 in outpatients [11,12]. In addition, the orally bioavailable but mutagenic nucleoside analog molnupiravir (MK-4482, EIDD-2801, Lagevrio) has also received FDA emergency use authorization; and early treatment significantly reduced the risk of hospitalization or death in at-risk, unvaccinated adults with COVID-19 [13]. Even more promising, the newly approved 3CLpro inhibitor paxlovid can be given orally and significantly reduces hospitalization time and death rates [14,15,16]. Besides these drugs, only monoclonal antibodies targeting the viral S protein, like regdanvimab or casirivimab/imdevimab, or the receptor for IL-6 (tocilizumab) are approved by the EMA to therapeutically treat COVID-19 patients. However, one serious problem associated with antibodies targeting the viral S protein is the emergence of resistance mutations, as it occurs in a number of circulating SARS-CoV-2 variants of concern (VOC) like the Delta (B1.617.2) or the Omicron (B1.1.529) BA.1 and BA.2 variants [17,18,19,20,21]. These drugs are all in need of adjustments due to new variants, especially the Omicron variant. Host-directed strategies to limit SARS-CoV-2 entry into target cells by either manipulating the ACE2 receptor or the S protein priming step represent promising target points for antiviral therapy. Since mice lacking TMPRSS2 do not exhibit a discernable phenotype [22], inhibition of TMPRSS2 activity or the reduction of TMPRSS2 expression may be an attractive way to prevent SARS-CoV-2 entry into host cells.

SMYD2 belongs to the five-member human SET and MYND (SMYD) domain-containing family of protein-lysine methyltransferases. The SET domain catalyzes the transfer of methyl groups to lysine residues on histone and non-histone proteins, whereas the MYND domain contains a zinc finger motif that mediates protein–protein interactions with target proteins. SMYD2-mediated modification of histones correlates with the enhanced transcription of genes involved in cell cycle regulation, DNA damage response, chromatin remodeling, and transcriptional regulation [23]. Moreover, methylation of non-histone proteins such as p53 or Rb can alter protein function [24,25]. SMYD2, which is essential for normal organismal development, was found to be dysregulated in cardiovascular disease and cancer, and overexpression often correlated with a lower survival rate [26,27,28,29,30,31,32]. Since SMYD2 represents a potential therapeutic target, various inhibitors have been developed. These inhibitors are potent cell-permeable and selective small molecules that either bind to the substrate channel of SMYD2 (AZ505, AZ506, LLY507), or address its methyl-lysine binding pocket (BAY598) [33,34,35].

Here, we could show for the first time that SMYD2 deficiency and its pharmacological inhibition by small molecules decreases TMPRSS2 expression on mRNA and protein levels. This downregulation was observed in multiple cell types including the SARS-CoV-2 target cell lines Caco-2 and Calu-3. In agreement with the downregulation of TMPRSS2, viral replication was significantly diminished in cells treated with the SMYD2 inhibitors AZ505 and BAY598, respectively. Of note, SMYD2 inhibition does not affect SARS-CoV-2 infection in Vero E6 cells, a cell line that lacks detectable TMPRSS2 protein levels. Therefore, we postulate that SMYD2 deficiency or inhibition blocks viral infection by reducing TMPRSS2 expression. Moreover, BAY598 displayed similar antiviral activity against the originally emerged WT virus and the Delta variant, demonstrating that downregulation of TMPRSS2 represents a promising tool to target the replication of newly emerging variants that otherwise escape neutralizing antibodies.

## 2. Materials and Methods

### 2.1. Biosafety

Cloning and preparation of pBSCoV2 bacmids containing the full-length SARS-CoV-2 genome were performed under biosafety level (BSL)-2 conditions. Virus recovery and infection experiments were conducted under BSL-3 conditions.

### 2.2. Cell Culture

In general, all cell lines were cultivated at 37 °C, 5% CO_2_, and 80% humidity and tested frequently for mycoplasma contaminations using the MycoAlert™ Mycoplasma Detection Kit (LT07-703, Lonza, Basel, Switzerland). Human embryonic kidney (HEK) 293T (ATCC^®^ CRL-3216™, ATCC, Manassas, VA, USA) and Vero E6 cells (85020206, Sigma-Aldrich, St. Louis, MO, USA) were cultured in Dulbecco’s Modified Eagle Medium (DMEM; 11500516, Thermo Fisher Scientific, Waltham, MA, USA) supplemented with 10% heat-inactivated fetal calf serum (FCS) (FBS-12A; Capricorn Scientific, Ebsdorfergrund, Germany), 2 mM GlutaMAX™ (35050061, Thermo Fisher Scientific), 25 mM HEPES (15630080, Thermo Fisher Scientific), and 50 µg/mL of gentamycin (1405-41-0, Serva Electrophoresis, Heidelberg, Germany). HT-29 (ATCC^®^ HTB-38™) cells were maintained in DMEM supplemented with 10% FCS and 1% penicillin/streptomycin (P433, Sigma-Aldrich, St. Louis, MO, USA). SMYD2 knockout cell lines were established as previously described [36]. Caco-2 cells (kindly provided by Konstantin Sparrer, University Hospital Ulm, Germany) were cultivated in DMEM, additionally containing 1× MEM Non-Essential Amino Acids Solution (11140050, Thermo Fisher Scientific). Calu-3 cells (kindly provided by Stephan Ludwig, University of Münster, Münster, Germany) were cultivated in Minimum Essential Medium (MEM; 15188319, Thermo Fisher Scientific) supplemented with 10% FCS, 2 mM GlutaMAX™, 25 mM HEPES, 1× MEM Non-Essential Amino Acids Solution, 1× sodium pyruvate (11360-038, Thermo Fisher Scientific), and 50 µg/mL of gentamycin. HEK293T-ACE2 and HEK293T-T7RNAP/N cells were generated and cultured as previously described [37].

### 2.3. LDH Cytotoxicity Assay

For SMYD2 inhibition, cells were treated with AZ505 (HY-15226), AZ506 (HY-134828), LLY-507 (HY-19313), or BAY598 (HY-19546) (all from Medchemexpress, Monmouth Junction, NJ, USA). These inhibitors were dissolved in DMSO. Cytotoxicity analysis was performed at 24 and 48 h post-treatment via the Lactate dehydrogenase (LDH) assay using the Cytotoxicity Detection Kit (11644793001; Sigma-Aldrich) according to the manufacturer’s protocol. Supernatants from cells cultured in medium with 0.1% Triton X-100 were used as the positive control and the cell culture medium was used as the negative control. Experiments were performed in triplicate wells at least two times and representative data are shown. The percentage of dead cells was calculated as follows:

$$\;\mathrm{cell}\;\mathrm{death}\;\left(\mathrm{LDH}\right)\;=\;\frac{{\left(\mathrm{LDH}\right)}_{\mathrm{sample}}-{\left(\mathrm{LDH}\right)}_{\mathrm{Negative}\;\mathrm{control}}}{{\left(\mathrm{LDH}\right)}_{\mathrm{Positive}\;\mathrm{control}}-{\left(\mathrm{LDH}\right)}_{\mathrm{Negative}\;\mathrm{control}}}\times 100$$

### 2.4. Cloning and Reconstitution of recSARS-CoV-2_d6-YFP_Spike Delta

Delta variant mutations in patient samples were first detected by next-generation sequencing upon positive RT-qPCR results using the Novaplex™ SARS-CoV-2 Variants VII Assay (Seegene, Düsseldorf, Germany). Total RNA isolated from the patient’s sample was utilized as a template for cDNA synthesis using the NEB LunaScript^®^ RT SuperMix Kit (E3010, New England Biolabs (NEB), Frankfurt am Main, Germany). For library preparation, the NEBNext^®^ ARTIC SARS-CoV-2 Library Prep Kit (E7650, NEB) was used. Sequencing was performed with the MiSeq system with the MiSeq Reagent Kit, v2 (300 cycles) (Illumina, San Diego, CA, USA). Sequences were analyzed with CLC Genomics Workbench 21 (Qiagen Aarhus A/S, Aarhus, Denmark). The patient sample used as a template for spike amplification was classified as the B.1.617.2 lineage. The isolate contained all characteristic mutations in the spike protein, with additional W152R and A222V mutations. The cDNA was used as a template for the spike amplification with spike-specific primers (forward: 5′-TTAACAACTAAACGAACAATGTTTGTT-3′, reverse: 5′-ACAAATCCATAAGTTCGTTTATGTGT-3′; IDT). The primer sequences include overhangs complementary to the pBSCoV2 vector, which contains the full-length SARS-CoV-2 genome [37]. The vector with a deletion in the original spike sequence was generated by insertion of a kanamycin resistance cassette instead of the spike gene using lambda-based Red recombination, as previously described [37]. The kanamycin cassette was removed by restriction digestion with SacII. The previously amplified spike sequence, then, was assembled with the linearized backbone using the NEBuilder HiFi DNA Assembly Cloning Kit (E5520, NEB) according to the supplier’s protocol. Correctly assembled clones were identified by restriction digestion and the Delta-specific spike mutations were confirmed by Sanger sequencing. Next-generation sequencing was applied to validate the integrity of the full-length SARS-CoV-2 genome. The recombinant reporter virus was recovered and titrated as previously described [37].

### 2.5. Infection Experiments

Caco-2, Calu-3, or Vero E6 cells were seeded in 96-well plates and pre-treated with the respective inhibitor at the indicated concentrations for 24 h. The cell culture medium was renewed with the same amount of inhibitor prior to infection. For infection experiments, the clinical isolate MUC-IMB-1 or different recombinant SARS-CoV-2 reporter viruses that have been described and characterized previously [37] were used. The cells were infected at an MOI of 0.005 and analyzed for virus production at 30 h post-infection. Infection rates were determined by immunostaining of viral spike protein expression, reporter expression or luciferase assay. To quantitatively evaluate stained plates, a Victor X4 multilabel reader (Perkin Elmer, Waltham, MA, USA) was used. The percentage of viral replication was assessed relative to solvent-treated cells. The 50% effective concentration (EC_50_) was determined by nonlinear four-parameter curve fitting using Graphpad Prism (v6, Graphpad Holdings LLC, San Diego, CA, USA). Experiments were repeated at least three times and representative results are shown.

### 2.6. Detection, Quantification and Analysis of Infected Cells

To determine the amount of infected cells upon inhibitor treatment, in-cell immunostaining and luciferase assays were used as previously described [37]. For the visualization of intracellular viral antigens, cells were stained with a mouse monoclonal antibody against the viral spike protein (mAB-S TRES-VI6.18 [38]) and Alexa488-conjugated secondary antibody (A11029, Thermo Fisher Scientific). Gaussia luciferase activity was quantified from heat-inactivated cell culture supernatants using Coelenterazine (102171, PJK Biotech, Kleinblittersdorf, Germany) as substrate.

### 2.7. Plaque Assay

Caco-2 cells were seeded in 12-well plates in confluent monolayers and pre-treated with BAY598 at the indicated concentrations for 24 h. The cells were infected with a 1:10,000 dilution of the recSARS-CoV-2_d6-YFP reporter virus (d6-YFP) [37]. After 2 h, the inoculum was removed and the cell culture medium containing the indicated concentration of BAY598 and 1.5% Avicel RL-591 (IFF Nutrition & Biosciences, Oestgeest, The Netherlands) was applied to the cells. Two days post-infection, the overlay was removed, cells were washed with PBS and fixed with 4% PFA in PBS. To visualize YFP-expressing plaques, an Advanced Fluorescence Imager (Intas Science Imaging, Göttingen, Germany) was used.

### 2.8. Western Blot Analysis

Tissue or cell extracts were prepared in RIPA lysis buffer (89900, Thermo Fisher Scientific) supplemented with protease and phosphatase inhibitor (A32961, Thermo Fisher Scientific). Lysates were cleared by centrifugation. Supernatant proteins were mixed with LDS Sample Buffer (NP0007, Thermo Fisher Scientific), separated by SDS-PAGE and transferred to nitrocellulose membranes (1704270, BioRad, Hercules, CA, USA). Membranes were probed with the following primary antibodies: anti-SMYD2 (sc-393827, Santa Cruz Biotechnology, Dallas, TX, USA), anti-TMPRSS2 (ab109131, Abcam, Cambridge, UK), and HRP-linked β-actin (ab49900, Abcam).

### 2.9. RNA Extraction and Quantitative Real-Time PCR

The peqGOLD RNA Kit (732-2868, Peqlab, Erlangen, Germany) was used to extract total RNA from tissues or cultured cells according to the manufacturer’s protocol. RNA was reverse transcribed into cDNA using the SCRIPT cDNA Synthesis Kit (PCR-5112, Jena Bioscience, Jena, Germany). Quantitative real-time PCR was performed with specific QuantiTect Primer assays (Qiagen, Hilden, Germany). *HPRT* or *ACTB* were used for normalization. Experiments were performed at least two times and representative data are shown.

### 2.10. RNA Sequencing

RNA extraction was done as mentioned above. After quality control, RNA samples were sequenced using an Illumina platform generating paired-end reads. Mapping on the reference genome and quantification was performed with STAR (2.7.0d) and feature counts (v1.6.4), respectively. Deseq2 (1.24.0) was used for the analysis of expression profiles of the different groups. In-house bioinformatics tools were used for enrichment, clustering, and other analyses.

### 2.11. Immunofluorescence Staining

Cells were grown on 8-well chamber slides with around 80% confluence (80841, Ibidi, Gräfelfing, Germany). After treatments, cells were fixed with 4% PFA and permeabilized with 0.1% Triton X-100. First antibodies were incubated overnight and fluorescence signals were visualized by confocal laser scanning microscopy (Leica TCS SP5 equipped with a 63 × 1.4 HCX PL APO CS oil immersion objective; Leica Microsystems, Wetzlar, Germany) and Leica Application Suite Advanced Fluorescence (v2.6.0, Leica Microsystems).

## 3. Results

### 3.1. SMYD2 Deficiency or Inhibition Results in the Downregulation of TMPRSS2 in HT-29 Cells

**Figure 1:**
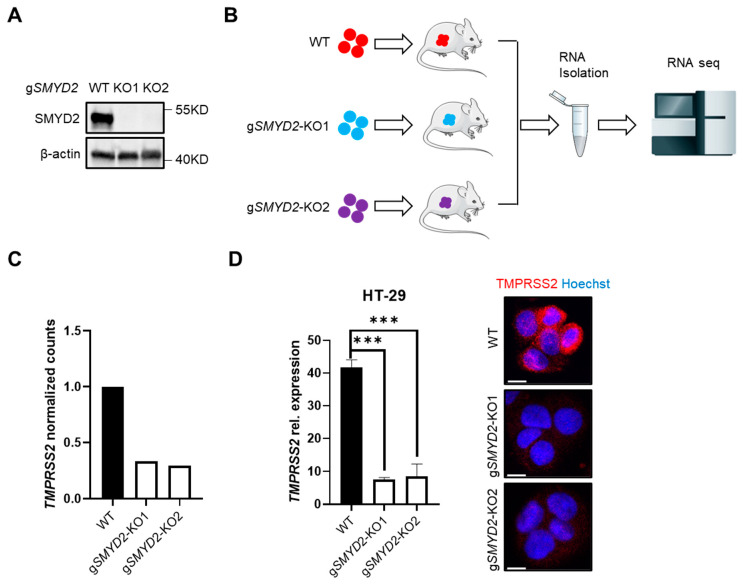
**SMYD2 deficiency decreases TMPRSS2 expression in HT-29 cells.** (**A**) Immunoblot analysis of SMYD2 in lysates of WT and two clones of CRISPR/Cas9-directed *SMYD2* knockout HT-29 cells. β-actin served as a loading control. (**B**) The workflow of RNA-seq analysis on HT-29 xenografts. (**C**) RNA-seq analysis of *TMPRSS2* expression (normalized counts) in HT-29 xenografts. (**D**) qPCR analysis (**Left panel**) or immunofluorescence staining (**Right panel)** of TMPRSS2 in cultured WT and *SMYD2* knockout HT-29 cells. Experiments were performed twice and representative data are shown. *** *p* < 0.001. Scale bar, 10 µm.

In the context of a project aimed to study the role of SMYD2 in cancer [36], SMYD2-sufficient and SMYD2-deficient HT-29 cells (Figure 1A), a human colorectal cancer cell line that has been widely used for many years, were implanted into Rag1^−/−^ mice via subcutaneous injection, and RNA-seq analysis of HT-29 xenografts was performed (Figure 1B). Interestingly, RNA-seq data analyses revealed that transcript levels of TMPRSS2 were significantly lower in both clones of SMYD2-deficient cells as compared to WT cells (Figure 1C). RT-PCR and immunofluorescence staining further confirmed the highly significant reduction of TMPRSS2 expression on mRNA and protein levels in SMYD2-deficient HT-29 cells under cultured conditions (Figure 1D).

**Figure 2:**
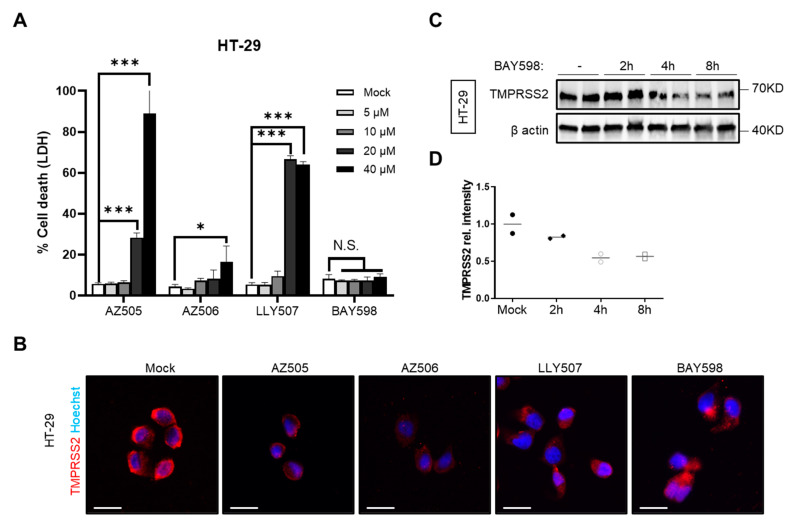
**SMYD2 inhibition decreases TMPRSS2 expression in cultured HT-29 cells.** (**A**) LDH release assay from HT-29 cells stimulated with increasing concentrations of AZ505, AZ506, LLY507, or BAY598 for 24 h. Experiments were performed two times and representative data of one experiment are shown. * *p* < 0.05 and *** *p* < 0.001. N.S., not significant. (**B**) Immunofluorescence staining of TMPRSS2 in HT-29 cells stimulated with vehicle (mock), 10 µM AZ505, 10 µM AZ506, 10 µM LLY507, or 20 µM BAY598 for 8 h. Scale bar, 10 µm. (**C**) Immunoblot analysis of TMPRSS2 in lysates of HT-29 cells at different time points upon treatment with 20 µM BAY598. β-actin served as a loading control. (**D**) Densitometric analysis of TMPRSS2 expression.

As therapeutic inhibition of SMYD2 gene expression is challenging, we strived to find a safe way to pharmacologically inhibit the function of SMYD2. Therefore, four different commercially available SMYD2 inhibitors, namely AZ505 [34], AZ506 [39], LLY507 [35], and BAY598 [33], were analyzed regarding their compatibility with our cell culture settings and their capacity to downregulate TMPRSS2. First, we determined the cytotoxicity via LDH release in HT-29 cells (Figure 2A). While AZ505, AZ506 and LLY507 showed cytotoxic effects at 20 µM after an incubation time of 24 h, BAY598 did not affect cell viability upon application on HT-29 cells (Figure 2A). Immunofluorescence staining of TMPRSS2 revealed a decreased expression after an incubation time of 8 h with the respective inhibitor (Figure 2B). Moreover, Western blot analysis confirmed the downregulation of TMPRSS2 as soon as 4 h post-treatment with BAY598 (Figure 2C). Collectively, these data confirm that the pharmacological inhibition of SMYD2 reduces TMPRSS2 protein levels in cultured HT-29 cells.

### 3.2. Inhibition of SMYD2 Impedes SARS-CoV-2 Infection of Caco-2 Cells

**Figure 3:**
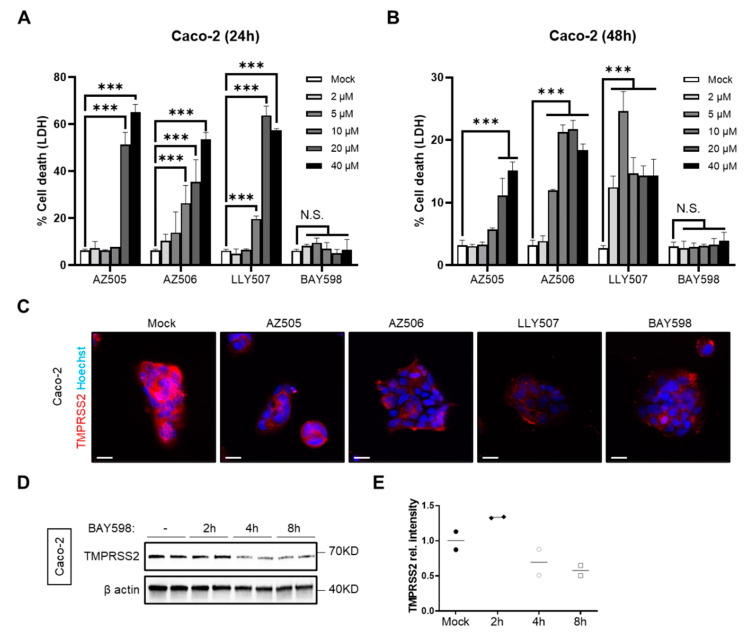
**SMYD2 inhibition decreases *TMPRSS2* expression in Caco-2 cells.** (**A**,**B**) LDH release assay from Caco-2 cells treated with increasing concentrations of AZ505, AZ506, LLY507, or BAY598 for 24 h (**A**) and 48 h (**B**). Experiments were performed two times and representative data are shown. *** *p* < 0.001. N.S., not significant. (**C**) Immunofluorescence staining of TMPRSS2 in Caco-2 cells stimulated with vehicle (mock), 10 µM AZ505, 10 µM AZ506, 10 µM LLY507, or 20 µM BAY598 for 8 h. Scale bar, 10 µm. (**D**) Immunoblot analysis of TMPRSS2 in lysates of Caco-2 cells stimulated with 20 µM BAY598 at different time points. β-actin served as a loading control. (**E**) Densitometric analysis of TMPRSS2 expression.

Since it has been shown that SARS-CoV-2 uses TMPRSS2 for priming the spike (S) protein to infect target cells [6], we were interested in the question of whether inhibition of SMYD2 could reduce TMPRSS2 levels in Caco-2 cells, a cell line that is frequently used for SARS-CoV-2 infection experiments. First, we applied the four different SMYD2 inhibitors on Caco-2 cells and analyzed their cytotoxicity via LDH release (Figure 3A,B). In agreement with the results obtained from HT-29 cells, treatment with AZ505, AZ506 and LLY507 caused cytotoxic effects at concentrations of 10 µM (AZ506 and LLY507) to 20 µM (AZ505) (Figure 3A), whereas incubation with BAY598 was well tolerated up to a concentration of 100 µM (Figure 3A and data not shown). To analyze the effect of SMYD2 inhibition on TMPRSS2 levels in Caco-2 cells, immunofluorescence (Figure 3C) and Western blot analyses (Figure 3D) were performed. Again, pharmacological inhibition reduced TMPRSS2 levels as early as 4 h post-treatment. Thus, SMYD2 inhibitors represent a suitable tool to reduce TMPRSS2 expression in Caco-2 cells.

**Figure 4:**
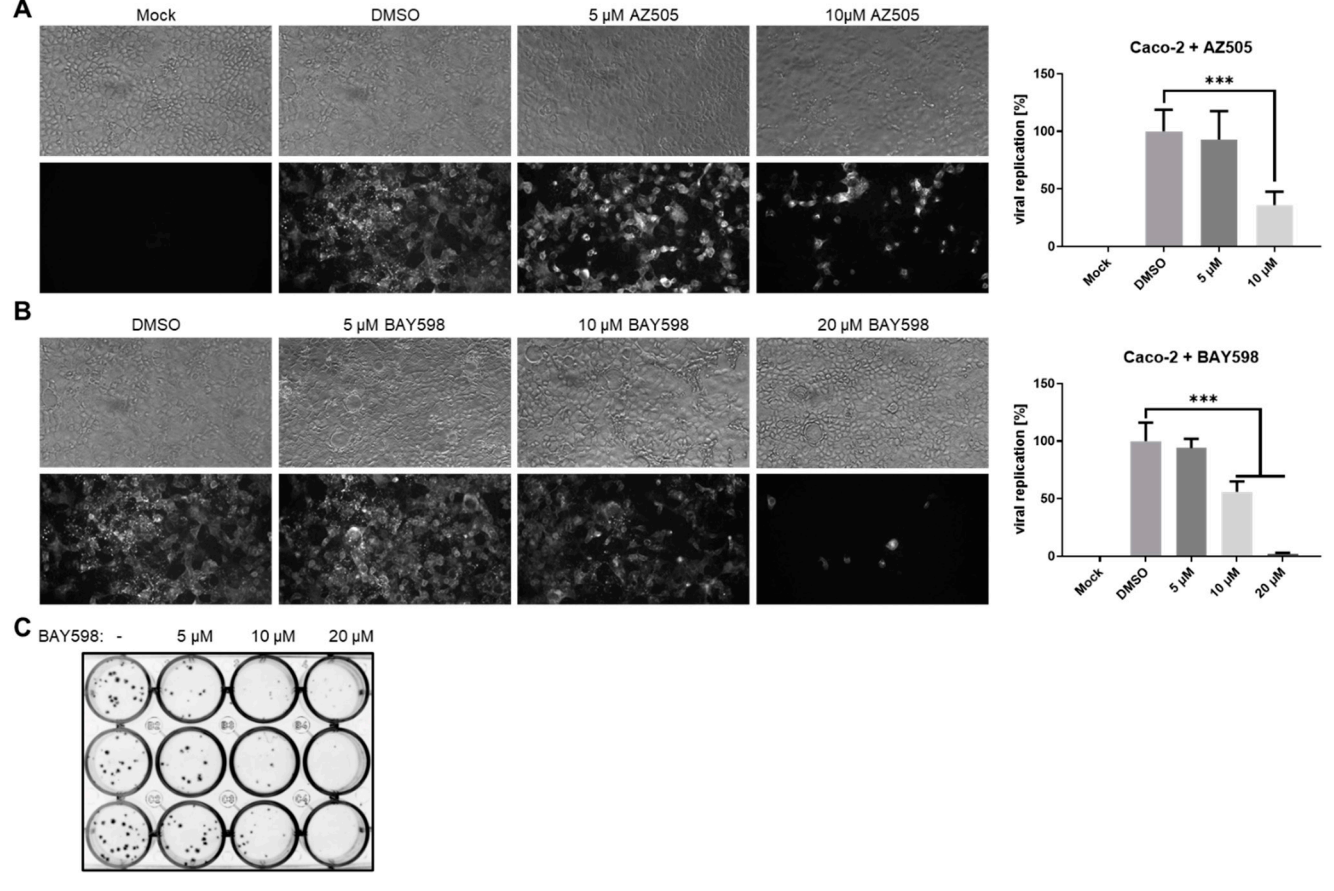
**SMYD2 inhibition decreases SARS-CoV-2 infection in Caco-2 cells.** (**A**,**B**) Antiviral effect of AZ505 (**A**) and BAY598 (**B**) in Caco-2 cells. Cells were treated with the indicated concentrations for 24 h prior to infection. The same amount of inhibitor was also applied during infection (MOI = 0.005). SARS-CoV-2 positive cells were visualized two days post-infection using a mAb against Spike and an Alexa488-conjugated secondary antibody under a Nikon Inverted Ts2-FL Eclipse microscope equipped with a 10x objective. The Alexa488 signal was quantified in a Victor X4 multilabel reader. Bar graphs represent mean values of eight infected wells ± SD. *** *p* < 0.001. N.S., not significant. One representative experiment out of three replicates is depicted. (**C**) Plaque assay of Caco-2 cells infected with a recombinant SARS-CoV-2 reporter virus expressing EYFP instead of the viral ORF6 gene (1:10,000 dilution) after treatment with the indicated concentrations of BAY598. EYFP-positive plaques were detected with a fluorescence imager two days post-infection. The experiment was repeated three times and representative results are shown.

Next, we aimed to characterize the effects of TMPRSS2 downregulation upon SMYD2 inhibition on viral replication. Due to the cytotoxicity data obtained from the LDH release assays (Figure 3A,B), we decided to focus on AZ505 and especially BAY598 for the following experiments. Caco-2 cells were pre-incubated with increasing concentrations of both inhibitors for 24 h prior to infection with the clinical SARS-CoV-2 isolate MUC-IMB-1 (B.1. by Pangolin nomenclature). During infection, the same amount of the respective inhibitor was supplied to the cell culture medium. After two days, the cells were fixed and incubated with the monoclonal anti-S antibody mAb-S TRES-VI6.18 [38]. Quantification of the fluorescence signal revealed a highly significant inhibition of viral replication in the presence of 10 µM AZ505 (Figure 4A). A highly significant block to viral infection was also observed after the treatment of Caco-2 cells with 10 µM and 20 µM BAY598 (Figure 3B). To confirm these results, we performed a plaque assay with a recombinant EYFP-expressing SARS-CoV-2 reporter virus (also B.1. by Pangolin nomenclature), in which the viral open reading frame (ORF) 6 is replaced by EYFP (deltaORF6-EYFP; d6-YFP) (Figure 4C). We could previously show that this reporter virus displays similar replication characteristics as the recombinant WT virus, which in turn behaves comparably to the clinical isolate in Caco-2 cells [37]. Again, cells were pre-treated with increasing concentrations of BAY598 for 24 h and the same amount of inhibitor was added to the overlay medium. After two days, the cells were fixed and EYFP-expressing plaques were directly visualized. In agreement with the data obtained with the S protein staining, increasing SMYD2 inhibitor concentrations clearly prevented viral plaque formation. Strikingly, in the presence of 20 µM BAY598, plaque formation was completely blocked. Taken together, these data demonstrate that the pharmacological inhibition of SMYD2 diminishes the replication of SARS-CoV-2 in Caco-2 cells. Since plaque formation is abolished in the presence of 20 µM BAY598, this finding underlines the block of virus entry into target cells, which most likely is the result of decreased TMPRSS2 expression.

**Figure 5:**
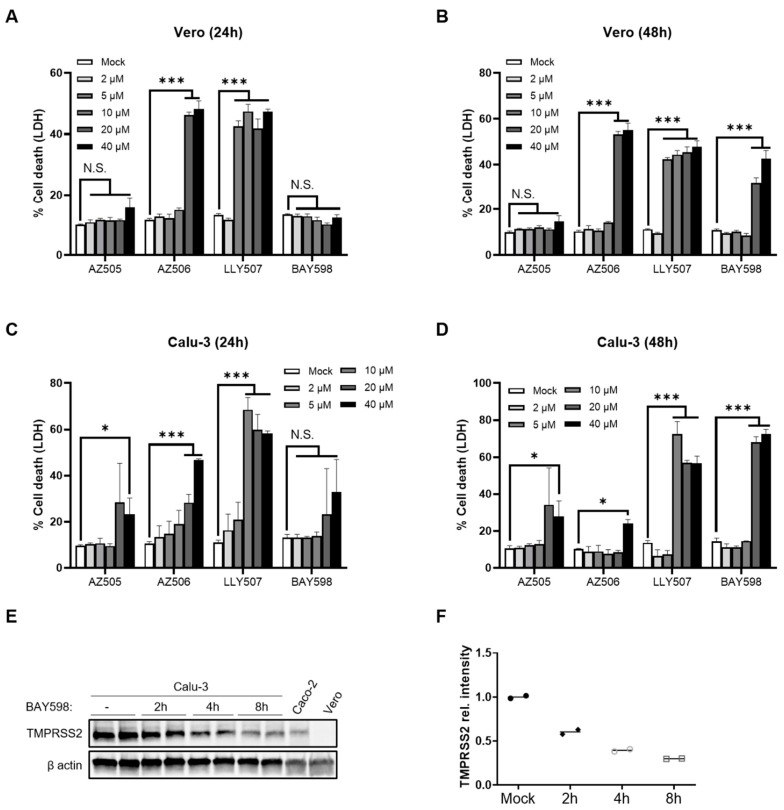
**SMYD2 inhibition decreases TMPRSS2 expression in Calu-3 cells.** (**A**–**D**) LDH release from Vero E6 (**A**,**B**) and Calu-3 cells (**C**,**D**) stimulated with the indicated concentrations of the respective inhibitor for 24 h (**A**,**C**) or 48 h (**B**,**D**). Experiments were performed two times and representative data are shown. *** *p* < 0.001. * *p* < 0.05. N.S., not significant. (**E**) Immunoblot analysis of TMPRSS2 in lysates of Calu-3 cells incubated with 20 µM BAY598 for the indicated time points. Caco-2 cells served as a positive control, while Vero E6 cells were used as a negative control. β-Actin was stained as the loading control. (**F**) Densitometric analysis of TMPRSS2 expression levels in Calu-3 cells.

To further elucidate the role of SMYD2 inhibition and TMPRSS2 downregulation during SARS-CoV-2 infection, we applied AZ505 and BAY598 onto Vero E6 and Calu-3 cells. Both cell lines are frequently used for SARS-CoV-2 experiments, but differ in their TMPRSS2 expression [6]. We initially determined the cytotoxic effects of the different SMYD2 inhibitors on both cell types by LDH assay after 24 and 48 h (Figure 5A–D). All inhibitors displayed higher cytotoxicity compared to Caco-2 cells, except AZ505, which did not show negative effects on cell viability in Vero E6 cells. Nevertheless, 10 µM of AZ505 and BAY598 were tolerated in both cell lines. Western blot analysis revealed higher TMPRSS2 expression in Calu-3 cells compared to Caco-2 cells (Figure 5E). In fact, TMPRSS2 was not detected in Vero E6 cells at all. In accordance with the data obtained previously for Caco-2 cells, incubation of Calu-3 cells with 20 µM BAY598 reduced the expression of TMPRSS2 as early as 2 h post-treatment (Figure 5E,F). This finding demonstrates that pharmacological inhibition of SMYD2 also decreases TMPRSS2 expression in Calu-3 cells.

### 3.3. Inhibition of SMYD2 Only Slightly Affects SARS-CoV-2 Infection in Calu-3 Cells

**Figure 6:**
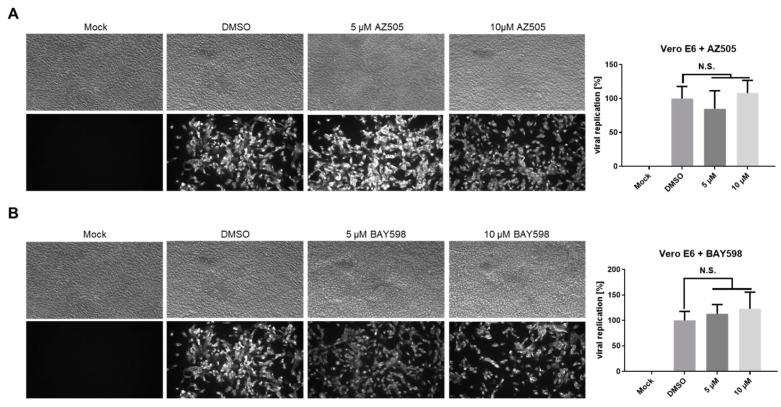
**SMYD2 inhibition does not affect SARS-CoV-2 infection of Vero E6 cells.** (**A**,**B**) Antiviral effect of AZ505 and BAY598 in Vero E6 cells. Cells were treated with the indicated concentrations for 24 h prior to infection. The same amount of inhibitor was also applied together with the virus (MOI = 0.005). SARS-CoV-2 positive cells were visualized two days post-infection using a mAb against Spike and an Alexa488-conjugated secondary antibody under a Nikon Inverted Ts2-FL Eclipse microscope equipped with a 10× objective. The Alexa488 signal was quantified in a Victor X4 multilabel reader. Bar graphs represent mean values of eight infected wells ± SD. N.S., not significant. One representative experiment out of three replicates is shown.

In the following experiments, we aimed to analyze the impact of SMYD2 inhibition on SARS-CoV-2 infection in Vero E6 and Calu-3 cells. Therefore, we pre-treated both cell types with AZ505 and BAY598 for 24 h, followed by infection with the clinical isolate MUC-IMB-1 for 48 h. S protein staining of fixed cells confirmed that the treatment of Vero E6 cells does not interfere with virus replication (Figure 6). This finding was expected since Vero cells do not express TMPRSS2 (Figure 5E) and underline the SMYD2 specificity of this approach. Taken together, these data suggested that SMYD2 may regulate SARS-CoV-2 infection via a TMPRSS2-dependent pathway.

**Figure 7:**
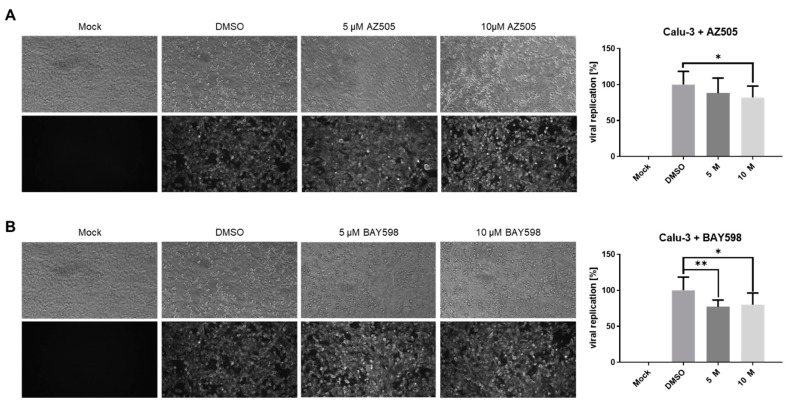
**Inhibition of SMYD2 in Calu-3 cells slightly impedes SARS-CoV-2 replication.** (**A**,**B**) Antiviral effect of AZ505 and BAY598 in Calu-3 cells. Cells were treated with the indicated concentrations for 24 h prior to infection. The same amount of inhibitor was also applied together with the virus (MOI = 0.005). SARS-CoV-2 positive cells were visualized two days post-infection using a mAb against Spike and an Alexa488-conjugated secondary antibody under a Nikon Inverted Ts2-FL Eclipse microscope equipped with a 10× objective. The Alexa488 signal was quantified in a Victor X4 multilabel reader. Bar graphs represent mean values of eight infected wells ± SD. ** *p* < 0.01. * *p* < 0.05. N.S., not significant. One representative experiment out of three replicates is shown.

Surprisingly, the presence of AZ505 or BAY598 only slightly decreased the percentage of virus-positive Calu-3 cells (Figure 7A). Quantification analysis based on the viral S protein staining showed around a 20% reduction in both cases. Taken together, the pharmacological inhibition of SMYD2 suppresses SARS-CoV-2 infection in the TMPRSS2-expressing lung cell line Calu-3. Notably, there might be cell type-specific differences in the magnitude of the inhibitory effect, since we observed a more pronounced restrictive effect in Caco-2 cells compared to Calu-3 cells.

### 3.4. BAY598 Similarly Inhibits Replication of Variants of Concern (VOC)

**Figure 8:**
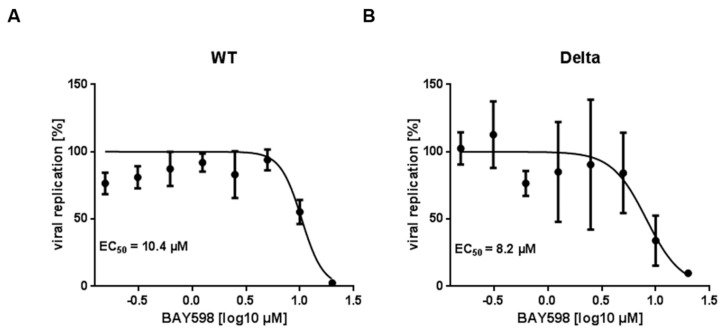
**Dose response of SARS-CoV-2 and the variants of concern, Delta and Omicron, to BAY598.** Caco-2 cells were pre-treated with increasing concentrations of BAY598 for 24 h. Afterwards, cells were infected with recombinant SARS-CoV-2 reporter viruses, either expressing WT spike (D614G) and a secreted gaussia luciferase instead of ORF7 (**A**), or the spike protein of the Delta variant and EYFP instead of ORF6 (**B**) (MOI = 0.005). In case of the GLuc reporter virus, cell culture supernatants were heat-inactivated after 30 h and analyzed for luciferase activity. To quantify EYFP-expression, cells were fixed 30 h post-infection and viral replication was assessed by reporter quantification. For both reporters, the percentage of viral replication was determined relative to DMSO-treated cells. The 50% effective concentration (EC_50_) was calculated by nonlinear four-parameter curve fitting using GraphPad Prism and is shown within the respective graph. Mean values of quadruplicates relative to the solvent control ± SD are depicted. One representative result out of four experiments is shown.

To precisely assess the antiviral activity of BAY598, which displays the lowest cytotoxicity (Figure 3B), we conducted infection experiments in Caco-2 cells and calculated the respective 50% effective concentration (EC_50_). We focused on the Delta (B.1.617.2) variant as an example of emerging variants of concern (VOCs). Given that the detection of clinical isolates with numerous mutations is often challenging, we decided to generate a recombinant reporter virus that, on the one hand, expresses EYFP instead of the viral ORF6 gene (d6-YFP), and on the other hand, the spike protein of the Delta variant. The virus was generated by Lambda-based Red recombination, as described previously [37], replacing the original spike sequence with a kanamycin resistance cassette flanked by unique endonuclease sites. After restriction digestion, the resulting backbone can be assembled with any PCR-amplified spike sequence of interest. We could previously show that the fluorescence readout of our recombinant marker virus d6-YFP is suitable for the assessment of antiviral activities since the results are consistent and comparable to the spike antigen staining of the recombinant full-length virus or the clinical isolate, as well as standard RT-qPCR analysis [37,40]. Moreover, we could also recently show that secreted gaussia luciferase (GLuc), which is expressed instead of the viral ORF7 protein (d7-GLuc), is also a suitable surrogate reporter to determine the EC_50_ values in Caco-2 cells [37]. To analyze the antiviral effect of BAY598, we infected Caco-2 cells, which were pre-treated with increasing concentrations of the inhibitor for 24 h, with the d7-GLuc reporter virus expressing the WT spike, or the d6-YFP reporter virus expressing the Delta spike. Calculated EC_50_ values of 10.4 µM for the WT (Figure 8A) and 8.2 µM for the Delta variant (Figure 8B) confirmed the antiviral potential of BAY598 against this VOC. Since their EC_50_ values are similar, lying in the lower micromolar range, this result demonstrates that inhibition of SMYD2 impedes SARS-CoV-2 infection independently of the spike sequence.

## 4. Discussion

More than two years ago, the highly transmissible severe acute respiratory coronavirus 2 (SARS-CoV-2) emerged and has been responsible for the ongoing COVID-19 pandemic, which is associated with more than 450,000,000 confirmed cases and over 6,000,000 deaths worldwide until March 2022. Tremendous efforts led to the rapid development of protective vaccines to protect people from SARS-CoV-2 infection. However, due to vaccination hesitancy and the initial shortage, a significant part of the population is infected or is facing the risk of infection [41,42]. Moreover, subjects who received two doses of vaccine or even additional booster shots can still be infected and develop symptomatic disease [43]. Even worse, several studies have accumulated evidence that the effectiveness of all approved SARS-CoV-2 vaccines is reduced against immune escape variants such as Delta and more recently, Omicron [17,18,19,20,21]. Therefore, effective anti-SARS-CoV-2 drugs are still urgently needed, especially oral bioavailable medications that can be distributed and administered early in the disease to prevent hospitalization, the need for intensive care, and death. Here, we identified SMYD2 as a new potential target for anti-SARS-CoV-2 treatment. Four different SMYD2 inhibitors have been used in our studies and two of them showed promising antiviral effects without significant cytotoxicity on cultured Caco-2 cells. Interestingly, BAY598, one of these two candidate inhibitors, did not show any negative effect on cell viability even at a very high concentration. In addition, oral administration of BAY598 has been shown to reduce SMYD2 function in an esophageal ex vivo model [33], suggesting that BAY598 administration may have therapeutic potential in COVID-19 treatment.

The SARS-CoV-2 S protein, a class-I viral fusion protein, is required for viral infection and its activation involves proteolytic processing [2,3]. Inhibition of proteolytic enzymes, including viral proteases and several host proteases involved in virus entry, have been identified as promising strategies to counteract SARS-CoV-2 infection. Targeting viral proteases, such as 3CLpro by paxlovid or GC376, demonstrated an antiviral effect against a broad spectrum of coronaviruses, at least in the in vitro models [16,44,45]. However, viral mutations may jeopardize the efficiency of these drugs in anti-SARS-CoV-2 treatment. Targeting host proteases, on the other hand, should be less subject to evasion, and show promising results [6,46,47]. TMPRSS2 is one of these host proteases. Our results clearly showed that SMYD2 deficiency or inhibition significantly decreased TMPRSS2 levels in multiple cell lines. Interestingly, SMYD2 inhibition showed strong antiviral effects only in TMPRSS2-sufficient Caco-2 cells but not TMPRSS2-deficient Vero cells, suggesting that SMYD2 may regulate SARS-CoV-2 infection via a TMPRSS2-dependent pathway. Compared to classical TMPRSS2 inhibitors, such as camostat mesylate and nafamostat, which inhibits its protease activity [6,47], SMYD2 inhibition targets TMPRSS2 protein levels. This may provide a more efficient way to block TMPSS2 function, or at least provide an alternative strategy regarding SARS-CoV-2 treatment. It would be interesting to test combination therapy involving SMYD2 inhibitors and drugs that modulate TMPRSS2 activity in order to achieve better antiviral effects with less cytotoxicity.

In our study, we tested the effects of SMYD2 inhibition on TMPRSS2 expression in HT-29 cells and Caco-2 cells, two human colorectal adenocarcinoma cell lines, and Calu-3 cells, one lung cancer cell line. The presence of SMYD2 inhibitors significantly decreased TMPRSS2 levels in all three cell lines. Surprisingly, unlike the results obtained from the experiments performed with the Caco-2 cells, which showed promising antiviral effects, SMYD2 inhibition only slightly decreased SARS-CoV-2 infection in Calu-3 cells. It has been shown that SARS-CoV-2 may use a pH-dependent but TMPRSS2-independent route to enter host cells [48]. Instead of using TMPRSS2, the virus uses cathepsin L, which can be activated under low pH conditions to prime viral fusion and penetration [48]. Other TMPRSS members, including TMPRSS11D and TMPRSS13, have also been shown to facilitate SARS-CoV-2 infection [49]. These alternative TMPRSS2-independent pathways may be involved to compensate for the effects caused by TMPRSS2 downregulation in Calu-3 cells, or Calu-3 cells are more likely to use the TMPRSS2-independent pathway compared to Caco-2 cells. This may explain why lower antiviral effects of SMYD2 inhibition have been observed in Calu-3 cells. Another possibility is that SMYD2 inhibition may not only downregulate TMPRSS2 expression but also SARS-CoV-2 Mpro or other mediators that are involved in SARS-CoV-2 infection. Those may only be targeted in Caco-2, but not in Calu-3 cells. Moreover, the protease activity of TMPRSS2 and other SARS-CoV-2 related proteases may also be affected by SMYD2 inhibition. Nevertheless, given the crucial role of TMPRSS2 expression in mediating SARS-CoV-2 infection, our study in different cell lines consistently demonstrated that SMYD2 inhibition downregulates TMPRSS2 expression. Thus, we believe that targeting SMYD2 posits great potential in anti-SARS-CoV-2 treatment. Notably, TMPRSS2 is also the activating protease for other coronaviruses, such as SARS-CoV and MERS, as well as other respiratory viruses, including influenza virus and parainfluenza viruses [50,51,52,53,54,55]. Therefore, downregulation of TMPRSS2 by targeting SMYD2 represents a promising tool to curtail further pandemic coronaviruses and possibly other respiratory viruses.

In summary, our study uncovered a novel role of SMYD2 in supporting SARS-CoV-2 infection. Inhibition of SMYD2 via commercially available drugs significantly decreased TMPRSS2 levels and decreased SARS-CoV-2 infection. Thus, one may speculate that SMYD2 serves as a new potential anti-SARS-CoV-2 target.

## Data Availability

Sequencing data have been deposited at Array Express under the accession number E-MTAB-11053.
